# Copper pollution exacerbates the effects of ocean acidification and warming on kelp microscopic early life stages

**DOI:** 10.1038/s41598-018-32899-w

**Published:** 2018-10-03

**Authors:** Pablo P. Leal, Catriona L. Hurd, Sylvia G. Sander, Evelyn Armstrong, Pamela A. Fernández, Tim J. Suhrhoff, Michael Y. Roleda

**Affiliations:** 1 Department of Botany, University of Otago, 479 Great King Street, Dunedin, 9016 New Zealand; 2 Institute for Marine and Antarctic Studies, University of Tasmania, 20 Castray Esplanade Battery Point, Hobart, 7004 Tasmania Australia; 3 Departamento de Repoblación y Cultivo, Instituto de Fomento Pesquero (IFOP), Balmaceda 252, Puerto Montt, Casilla 665 Chile; 4 NIWA/ University of Otago Research Centre for Oceanography, Chemistry Department, Union Place West, Dunedin 9016, New Zealand, Dunedin, 9016 New Zealand; 5 Marine Environment Study Laboratory, International Atomic Energy Agency, 4 Quai Antione 1er, 98000 Monaco, Monaco; 6 Centro i~mar, Universidad de Los Lagos, Camino a Chinquihue Km 6, Puerto Montt, Casilla 557 Chile; 7 ETH Zürich, Institute of Geochemistry and Petrology, Department of Earth Sciences, Clausiusstrasse 25, 8092 Zürich, Switzerland; 8 Norwegian Institute of Bioeconomy Research, Kudalsveien 6, 8027 Bodø, Norway; 9 The Marine Science Institute, College of Science, University of the Philippines Diliman, Quezon City, Philippines

## Abstract

Ocean warming (OW), ocean acidification (OA) and their interaction with local drivers, e.g., copper pollution, may negatively affect macroalgae and their microscopic life stages. We evaluated meiospore development of the kelps *Macrocystis pyrifera* and *Undaria pinnatifida* exposed to a factorial combination of current and 2100-predicted temperature (12 and 16 °C, respectively), pH (8.16 and 7.65, respectively), and two copper levels (no-added-copper and species-specific germination Cu-EC_50_). Meiospore germination for both species declined by 5–18% under OA and ambient temperature/OA conditions, irrespective of copper exposure. Germling growth rate declined by >40%·day^−1^, and gametophyte development was inhibited under Cu-EC_50_ exposure, compared to the no-added-copper treatment, irrespective of pH and temperature. Following the removal of copper and 9-day recovery under respective pH and temperature treatments, germling growth rates increased by 8–18%·day^−1^. The exception was *U*. *pinnatifida* under OW/OA, where growth rate remained at 10%·day^−1^ before and after copper exposure. Copper-binding ligand concentrations were higher in copper-exposed cultures of both species, suggesting that ligands may act as a defence mechanism of kelp early life stages against copper toxicity. Our study demonstrated that copper pollution is more important than global climate drivers in controlling meiospore development in kelps as it disrupts the completion of their life cycle.

## Introduction

The global climate is projected to change during the twenty-first century due mainly to anthropogenic combustion of fossil fuels and changes in land use^1^. In the marine environment, the projected future scenario includes a 4 °C increase in sea surface temperature and a reduction in pH from the current average of 8.10 to 7.74, phenomena known as ocean warming (OW) and ocean acidification (OA), respectively^1^. In addition, the hydrolysis of CO_2_ in seawater is increasing the concentration of H^+^, CO_2_, HCO_3_^−^, and decreasing the CO_3_^2−^ concentration^2^. However, the future scenario of OW and OA is not occurring in isolation from other anthropogenic activities that also threaten coastal environments at local levels^3^. For instance, in coastal environments, natural concentrations of copper (Cu^2+^) are low, but they are increasing due to human industrialization^4^. At high concentrations, copper becomes toxic, affecting metabolic processes of marine organisms. For example, >0.08 µM Cu negatively affect the completion of different life stages of brown macroalgae (species in the Order Fucales and Laminariales)^5^. The speciation and bioavailability of copper in seawater is highly dependent on seawater chemistry^6,7^. Metals such as Cu^2+^ can form inorganic complexes with CO_3_^2−^, OH^−^, and Cl^−^ ^6,7^, and organic complexes with organic ligands (L) such as thiols, exopolysaccharides and humic substances^8,9^. OA will reduce seawater CO_3_^2−^ concentrations and thus the stability of reaction constants in the formation of organic complexes: the toxic free ionic form of copper (i.e., speciation of Cu^2+^) in the oceans is thus predicted to increase by >50% by the end of the current century^6^. These changes in seawater chemistry have the potential to affect the physiological processes of marine organisms including macroalgae.

Coastal ecosystems from mid-latitudes to polar regions experience seasonal variations in environmental factors, including temperature and pH^10^, which are beyond the predictions by 2100 for the global ocean^11^. For example, daytime coastal seawater temperature can vary between 2–7 °C^12,13^ while diurnal seawater pH can vary by >1 unit due to macroalgal metabolism^14,15^. On the other hand, natural concentrations of copper in coastal seawater are generally low (0.008 and 0.050 µM)^16^, local human activities such as the production of industrial and domestic wastes, agricultural practices, copper mine drainage and usage of copper containing marine anti-fouling paint can result in local increases in copper concentrations above 3.0 µM^4,17,18^. Copper is an essential trace element for some biological functions in macroalgal physiology. For example, it forms part of the plastocyanin protein involved in photosynthetic electron transport and is a cofactor of the enzymes Cu/Zn-superoxide dismutase, cytochrome c oxidase, ascorbate oxidase, amino oxidase and polyphenol oxidase. However, at elevated concentrations, copper can be toxic to macroalgae and also a to a wide range of other marine organisms^4,19,20^.

Many organisms, including photosynthetic ones, can counteract the negative effects of high copper concentrations by the production of L^4,21–25^. L bind Cu, reducing its free ionic form (Cu^2+^) and weakly bound labile copper (Cu’) concentrations and thereby its toxicity. Thus, in a medium with an excess of copper, cells can transport the non-toxic ligand-bound copper (CuL) into or out of the cell across the plasma membrane for using or detoxifying copper, respectively^4^. The concentration and stability constants of L in solution (in seawater and culture media) can be indirectly determined by complexometric titration with copper^26^ or by using a kinetic approach^27,28^. In both cases, anodic or cathodic stripping voltammetry (ASV and CSV, respectively) are used as the detection method. The inorganic and labile organic complexes of copper (Cu’), measured using these voltammetric techniques, are considered bioavailable for biota, including macroalgae^26^. However, the structure of the L produced by macro- and microalgae are largely unknown^21,26^.

The life cycle of macroalgae consists of alternate microscopic and macroscopic stages. In kelps, microscopic meiospores (haploid spores resulting from meiosis) settle and develop into either male or female gametophytes. After fertilization, a diploid embryo is formed which grows to form the macroscopic adult^29^. Early life history stages of marine organisms are generally more sensitive to abiotic stress than their adult phase^30,31^ but studies on the effects of climate change on the microscopic phases of macroalgae are scarce^2,32,33^. The synergistic and additive toxic effects of copper under OA on early life stages of some marine invertebrates have been previously studied^34,35^. However, although macroalgal microscopic stages are highly sensitive to copper and hence have been widely used for ecotoxicity research^5,36^, the toxic effects of copper under increased temperature and/or OA conditions on early life stages have not been investigated.

Previous studies have shown that the independent and interactive effects of OA (pH 7.65) and OW (+4 °C) have little effect on the ontogenetic development of kelp meiospores^32,33^. This means that the completion of the life cycle from meiospore germination to sexual differentiation, and sexual reproduction to produce the next generation of adult sporophytes^37^ is unlikely to be compromised. Conversely, copper as a local environmental stressor was found to arrest sexual differentiation^5^, thus disrupting the completion of the life cycle. In this study, the interactive effects of seawater temperature (12 °C and 16 °C), pH_T_ (8.16 and 7.65) and copper pollution on the ontogenic development of meiospores of the native *M*. *pyrifera* and the invasive *U*. *pinnatifida*, from south-eastern New Zealand, were studied. The nominal copper concentrations used in this experiment correspond to the species-specific Cu-EC_50_ for meiospore germination of *M*. *pyrifera* (2.47 µM Cu_T_ = 157 µg L^−1^ Cu_T_) and *U*. *pinnatifida* (3.63 µM Cu_T_ = 231 µg L^−1^ Cu_T_)^5^. We hypothesized that the negative effect of copper on meiospore development (i.e., germination, germling growth, gametophyte production and sexual differentiation) will be greater under future climate change scenarios (e.g., Cu × OA, Cu × OW, and Cu × OA × OW). However, production of L may alleviate any negative effects of copper on meiospore development. Moreover, the capacity of germlings and gametophytes to recover from local environmental drivers by removing the copper treatment after 9 days was investigated under each climate change scenario for a further 9 days. This is the first study on the interactive effects of global climate change drivers (OA and OW) and a local driver (metal pollution) on the early life history stages of key marine forest-forming species, and their capacity to recover from local environmental pollution.

## Results

### Meiospore germination

**Figure 1:**
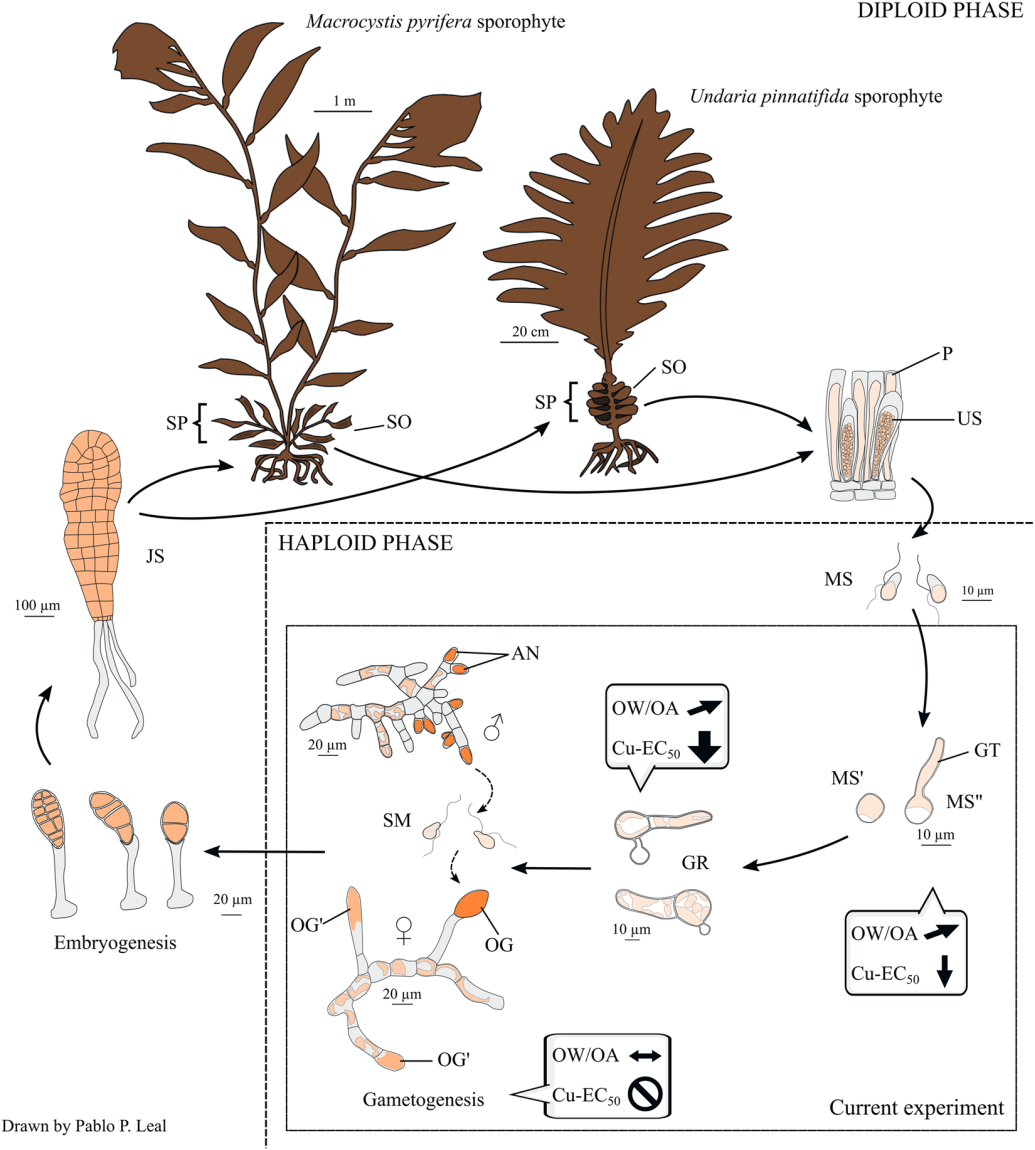
Summary of the main results of the current experiment (inner square). Dialogue boxes indicate the main effects of ocean warming (OW), ocean acidification (OA) and copper pollution (Cu-EC_50_) treatments on meiospore germination, germling growth rate and gametophyte development of *M*. *pyrifera* and *U*. *pinnatifida* (Order Laminariales). Left-right arrow (↔) indicates neutral effects, inclined arrow (↗) indicates slightly positive effects, downward arrow (↓) indicates negative effects (the thickness of only ↓ represents the magnitude of effects) and circle-backslash symbol (⦸) indicates that gametogenesis was inhibited. AN, antheridia; GR, germling; GT, germination tube; JS, juvenile sporophyte; MS, swimming meiospore; MS’, settled meiospore; MS”, germinated meiospore; OG, oogonium; OG’, oogonium in formation; P, paraphysis; SM, sperm; SO, sorus; SP, sporophyll; and US, unilocular sporangium. Drawings were made based on photomicrographs taken during this study.

**Figure 2:**
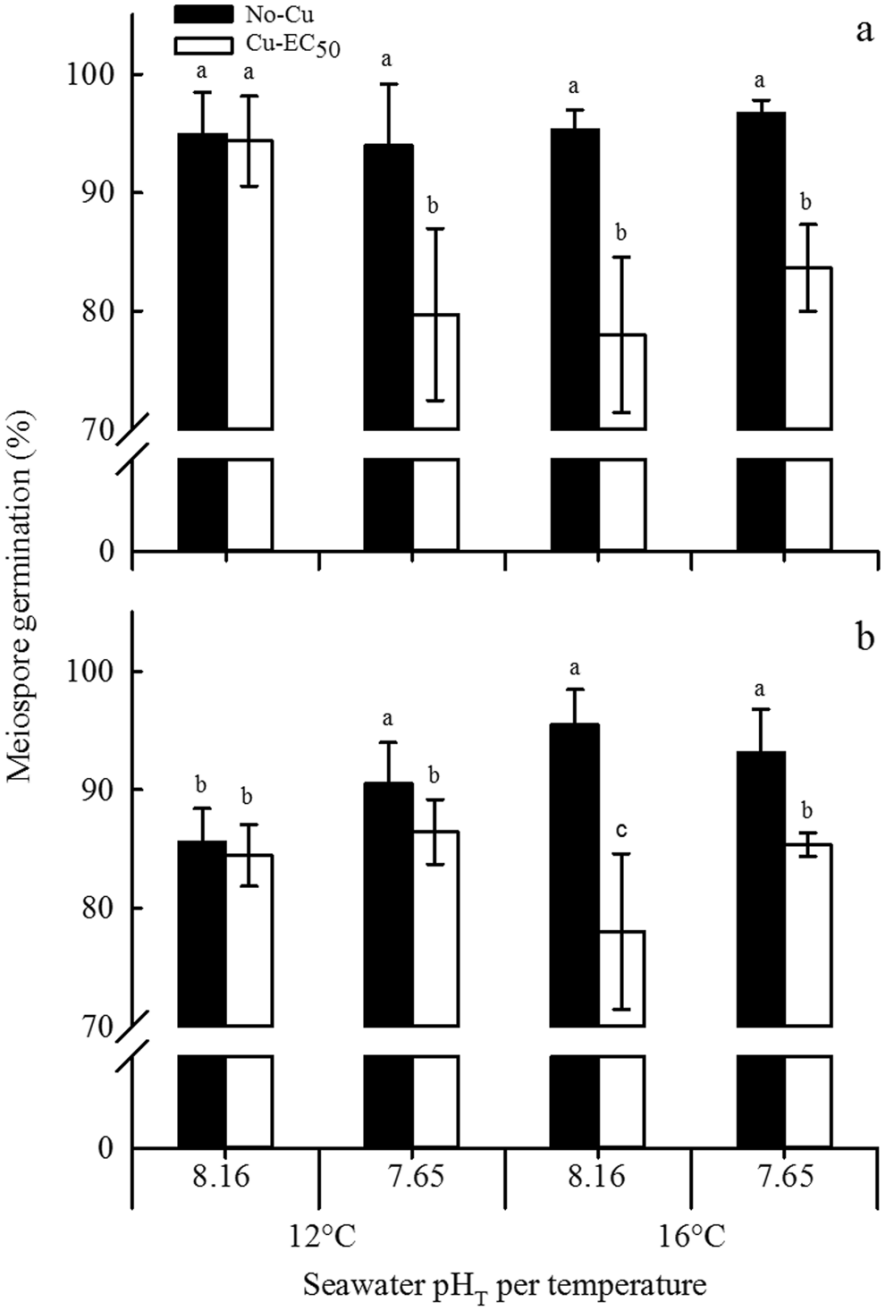
Percentage meiospore germination of (**a**) *M*. *pyrifera* and (**b**) *U*. *pinnatifida* after 6 days of culture in a factorial combination of two temperatures (12 and 16 °C), two pH (pH_T_ 7.65 and 8.16) and two copper (No-Cu, and Cu-EC_50_ = 2.36 and 3.62 µM Cu for *M*. *pyrifera* and *U*. *pinnatifida*, respectively) treatments. Bars represent mean ± SD (n = 4). Significant subgroups are grouped by the lowercase groups as a > b > c (Tukey, *P* < 0.05). Note that the y-axis has a break from 10 to 70% in both graphs.

After 6 days, the percentage of germinated meiospores (i.e., with visible germ tube, Fig. 1) was calculated for both species. OA and OW had no significant effect on the germination of meiospores of both *M*. *pyrifera* and *U*. *pinnatifida* (germination >85%) (Fig. 2). A significant (*P* < 0.001) detrimental effect of copper (i.e., 5–18% reduction) on germination of meiospores of both species was observed in all treatment combinations, except under ambient temperature and current pH (Fig. 2). The greatest (*P* < 0.05, Tukey test) additional effect of copper in the reduction of germination was observed at current pH and OW in *U*. *pinnatifida* (18%) (Fig. 2b).

### Germling growth rate

**Figure 3:**
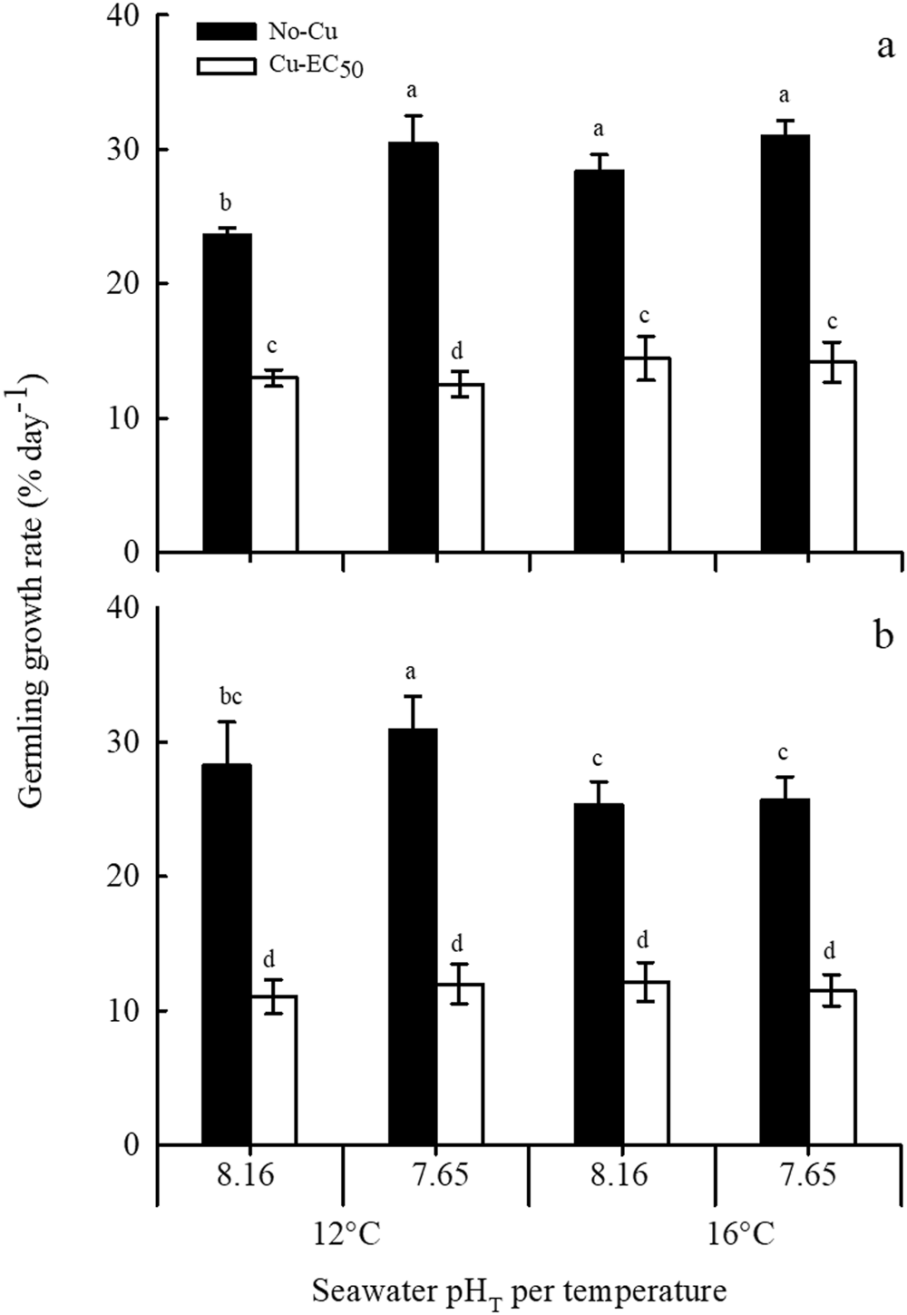
Growth rate of germlings of (**a**) *M*. *pyrifera* and (**b**) *U*. *pinnatifida* after 12 days of cultivation in a factorial combination of two temperatures (12 and 16 °C), two pH (pH_T_ 7.65 and 8.16) and two copper (No-Cu, and Cu-EC_50_ = 2.36 and 3.62 µM Cu for *M*. *pyrifera* and *U*. *pinnatifida*, respectively) treatments. Bars represent mean ± SD (n = 4). Significant subgroups are grouped by the lowercase groups as a > b > c > d (Tukey, *P* < 0.05).

The growth rate of sexually ambiguous germlings (Fig. 1) was calculated for both species (1–12 d for germlings under No-Cu and 1–9 d for those under Cu-EC_50_; Supplementary Fig. S1). When taken as individual factors, OW and OA significantly (*P* < 0.005) increased the growth rate of *M*. *pyrifera* germlings (7–20% increase) (Fig. 3a). In contrast, the growth of *U*. *pinnatifida* germlings was not affected by OW or OA (*P* > 0.05) (Fig. 3b). An additional significant (*P* < 0.001) effect of copper, causing a reduction in germling growth rate of *M*. *pyrifera* (by 46–63%) and *U*. *pinnatifida* (by 56–68%) was observed in all treatment combinations (Fig. 3).

### Gametophyte size

**Figure 4:**
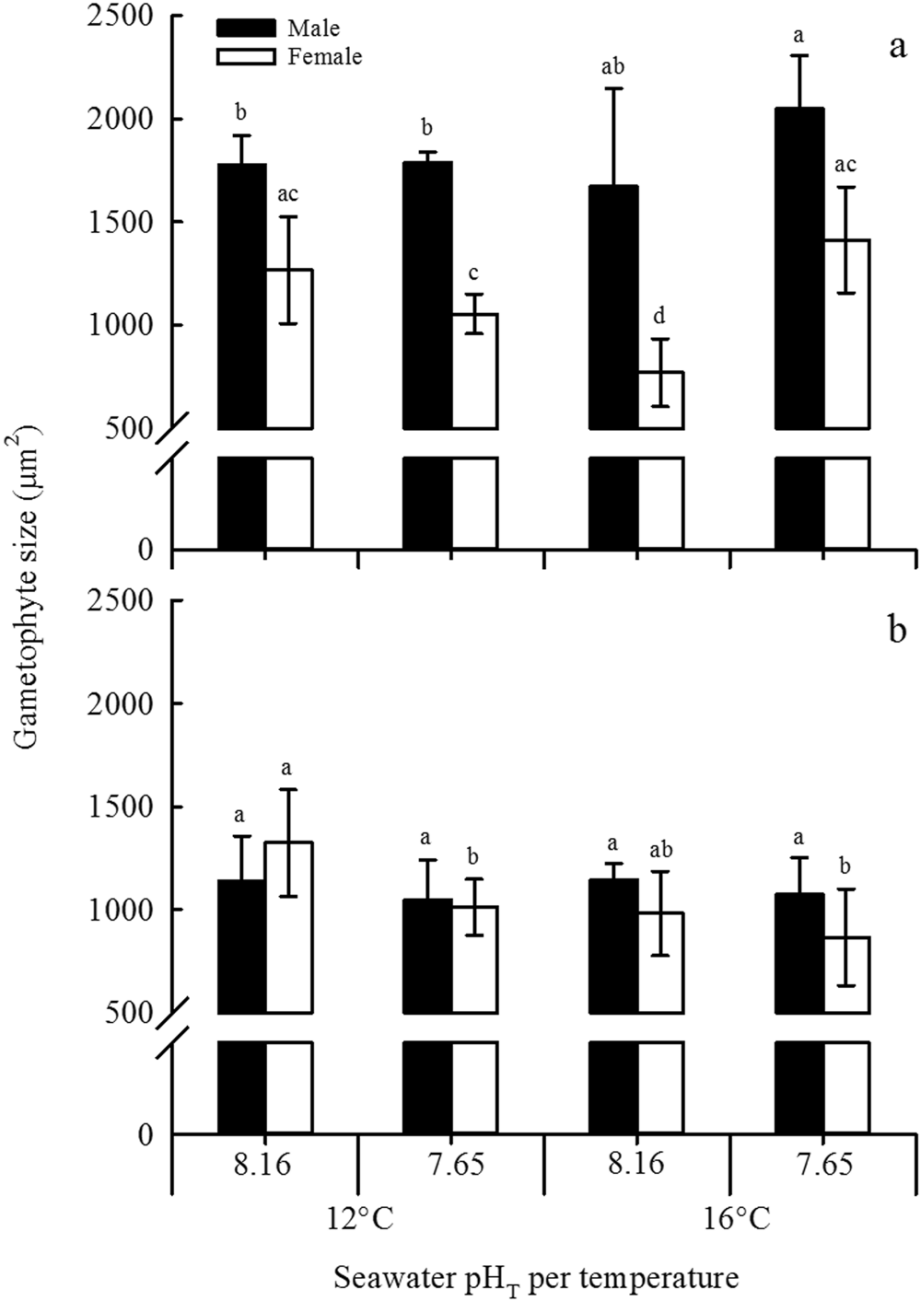
Size of male and female gametophytes of (**a**) *M*. *pyrifera* and (**b**) *U*. *pinnatifida* at the 15^th^ day of cultivation in a factorial combination of two temperatures (12 and 16 °C) and two pH (pH_T_ 7.65 and 8.16) treatments under control (no-copper addition) treatment. No data is available for the Cu-EC_50_ treatment as sexual differentiation was inhibited by copper exposure. Bars represent mean ± SD (n = 4). Significant subgroups are grouped by the lowercase groups as a > b > c > d (Tukey, P < 0.05). Note that the y-axis has a break from 100 to 500 µm^2^.

Sexual differentiation of gametophytes (Fig. 1) occurred at the 15^th^ day of culture for both species under No-Cu conditions. Gametophyte development and sexual differentiation were significantly (*P* < 0.001) inhibited by copper exposure (Fig. 4). In the No-Cu treatment, gametophytes of *M*. *pyrifera* grew significantly bigger (18% increase for males and 46% increase for females; *P* < 0.05, Tukey test) under OA at 16 °C, and males were significantly (*P* < 0.001) bigger (29–54%) than females under all pH and temperature combinations (Fig. 4a). In contrast, only the size of female gametophytes of *U*. *pinnatifida* was significantly (*P* < 0.05, Tukey test) reduced (24%) by OA at 12 °C compared to those at pH_T_ 8.16 at 12 °C (Fig. 4b).

### Gametophyte sex ratio

After 15 days, the sex ratio of sexually differentiated gametophytes under No-Cu treatment of both species varied between 0.47 and 0.53 but was not significantly affected by single factors nor their interactions (Supplementary Fig. S2).

### Germling growth rate during the recovery period

**Figure 5:**
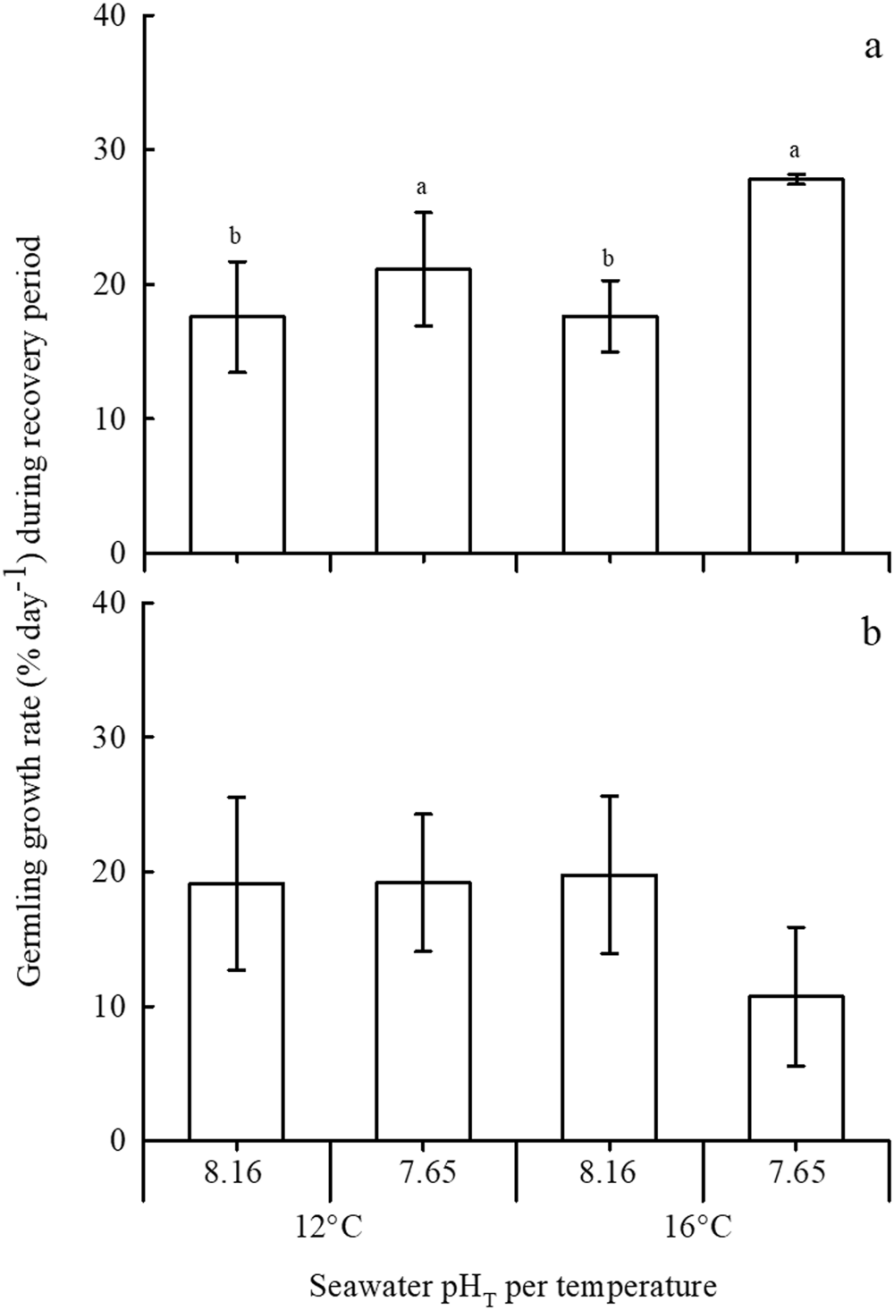
Growth rate for germlings of (**a**) *M*. *pyrifera* and (**b**) *U*. *pinnatifida* during recovery from copper Cu-EC_50_ exposure at corresponding temperatures (12 and 16 °C) and pH_T_ (7.65 and 8.16). Growth rate was calculated from day 12 to 18. Bars represent mean ± SD (n = 4). Significant subgroups are grouped by the lowercase groups as a > b (Tukey, P < 0.05).

The growth rate of sexually ambiguous germlings (Fig. 1) during the recovery period (after stopping copper addition to the media of the Cu-EC_50_ treatments on day 9) was calculated from day 12 to 18 for both species. In *M*. *pyrifera*, recovery of germling growth rate was significantly (*P* = 0.026) 7 and 25% greater in OA conditions compared to the current pH_T_ treatment at 12 or 16 °C, respectively (Fig. 5a). In contrast, OW, OA and their interactions did not significantly affect germling growth rate of *U*. *pinnatifida* during recovery (Fig. 5b). Moreover, recovering germlings of both kelps did not differentiate into male or female gametophytes by the end of the experimental period (day 18). When comparing the growth rate during the recovery period (Fig. 5) with that during the Cu-EC_50_ exposure (Fig. 3), the germling growth rate of *M*. *pyrifera* significantly (*P* < 0.001) increased. That increase in growth rate of *M*. *pyrifera* was 29–33% greater under pH_T_ 7.65 and 12 °C compared to pH 8.16 and 16 °C (*P* < 0.05, Tukey test). There were no statistical differences between growth rate during copper exposure and recovery for *U*. *pinnatifida* germlings under all pH and temperature combinations.

### Total dissolved copper (Cu_T_) concentrations

At the nominal copper concentrations corresponding to the Cu-EC_50_ for *M*. *pyrifera* (2.36 µM Cu) and *U*. *pinnatifida* (3.62 µM Cu), Cu_T_ concentrations measured in the fresh media were 2.51 and to 3.84 µM Cu_T_, respectively (Table 1). During the first 9 days of culture, when the media corresponding to the different treatments was renewed every third day, the dissolved concentration of Cu_T_ was reduced by 64–71% for *M*. *pyrifera* and by 66–72% for *U*. *pinnatifida*, under all temperature and pH treatment combinations_._ Although copper was not added on the 12^th^ and 15^th^ day of culture, Cu_T_ was still detectable in the culture media, but at much lower concentrations compared to the period when copper-treatment media was renewed every 3 days (Table 1).

**Table 1:** Copper speciation (concentrations of total dissolved copper, Cu_T_; labile copper, Cu’; and Cu-binding ligands, L) in culture medium of *M*. *pyrifera* and *U*. *pinnatifida* under two temperatures (12 and 16 °C), two pH (pH_T_ 7.65 and 8.16) in the respective Cu-EC_50_ concentration.

Species	Culture day	Cu_T_ in fresh culture medium (µM)	12 °C								16 °C							
			pH_T_ 8.16				pH_T_ 7.65				pH_T_ 8.16				pH_T_ 7.65			
			Cu_T_ (µM)	Cu’ (µM)	Cu_T_: Cu’ ratio	L (nM)	Cu_T_ (µM)	Cu’ (µM)	Cu_T_: Cu’ ratio	L (nM)	Cu_T_ (µM)	Cu’ (µM)	Cu_T_: Cu’ ratio	L (nM)	Cu_T_ (µM)	Cu’ (µM)	Cu_T_: Cu’ ratio	L (nM)
*M. pyrifera*	3	2.51	0.728 ± 0.025	0.189	3.9	bdl	0.677 ± 0.033	0.165	4.1	bdl	0.744 ± 0.014	0.178	4.2	bdl	0.740 ± 0.022	0.201	3.7	bdl
	6	2.51	0.890 ± 0.190	0.263	3.4	bdl	0.703 ± 0.025	0.203	3.5	bdl	0.845 ± 0.028	0.228	3.7	bdl	0.768 ± 0.008	0.263	2.9	bdl
	9	2.51	0.799 ± 0.014	0.222	3.6	bdl	0.749 ± 0.005	0.242	3.1	bdl	0.744 ± 0.011	0.211	3.5	bdl	0.812 ± 0.001	0.225	3.6	bdl
	12	0	≤0.047	0.014	≤3.3	9.7	≤0.047	0.016	≤3.0	11.1	≤0.047	0.016	≤3.0	10.3	≤0.047	0.013	≤3.7	2.5
	15	0	≤0.047	0.005	≤10.0	6.6 (5.2)	≤0.047	0.005	≤10.0	4.7 (2.4)	≤0.047	0.003	≤14.9	3.3 (3.5)	≤0.047	0.005	≤10.0	6.6 (2.4)
*U. pinnatifida*	3	3.84	1.158 ± 0.003	0.318	3.6	bdl	1.171 ± 0.017	0.326	3.6	bdl	1.174 ± 0.035	0.252	4.7	bdl	1.061 ± 0.052	0.290	3.7	bdl
	6	3.84	1.158 ± 0.020	0.275	4.2	bdl	1.150 ± 0.005	0.316 ± 0.044	3.6	bdl	1.133 ± 0.009	0.291	3.9	bdl	1.212 ± 0.085	0.305	4.0	bdl
	9	3.84	1.167 ± 0.005	0.346	3.4	bdl	1.190 ± 0.001	0.268	4.4	bdl	1.185 ± 0.068	0.458	2.6	bdl	1.278 ± 0.002	0.365	3.5	bdl
	12	0	0.127 ± 0.022	0.039	3.2	25.5	0.098 ± 0.019	0.035	2.8	20.5	0.168 ± 0.006	0.057	3.0	12.7	0.151 ± 0.035	0.033	4.6	6.6
	15	0	≤0.047	0.011	≤4.3	8.3 (3.5)	≤0.047	0.009	≤5.0	8.3 (3.5)	≤0.047	0.008	≤6.0	8.9 (5.0)	≤0.047	0.011	≤4.3	10.4

Copper was added to the culture media of the Cu-EC_50_ treatment for the first 9 days of culture. Cu_T_ was measured from two replicates and Cu’ data correspond to non-replicated samples, except for *U*. *pinnatifida* at 12 °C and pH_T_ 7.65 on day 6, where good reproducibility was shown for n = 4. L concentrations on day 3–9 were ≪Cu’ and therefore below the detection limit (bdl). Only when Cu additions were stopped did L become detectable, although still <Cu’. Please note that L is given in nM Cu-binding capacity. Values of L at day 15 in parenthesis are the L concentrations in the control culture (i.e. no Cu stress).

### Labile copper (Cu’) concentrations

During the first 9 days of culture, Cu’ concentrations varied between 0.157 and 0.268 µM in the culture media of *M*. *pyrifera* and between 0.252 and 0.456 µM in the culture media of *U*. *pinnatifida*, under all temperature and pH treatment combinations_._ Although copper was not added on the 12^th^ and 15^th^ day of culture, Cu’ was detectable but at much lower concentrations compared to the copper-added period (Table 1).

### Copper-binding ligand (L) concentration

Due to the high Cu_T_ in the media during days 0–9, L concentrations present in the cultures were undetectable. After the removal of Cu from the media (day 9), L was detected on day 12 and 15 (Table 1). At 12 °C and under both pH treatments, L release was higher (by >40%) on day 12 than day 15 in the culture media of both *M*. *pyrifera* and *U*. *pinnatifida*. At 16 °C, L release in the culture media of both kelps under pH 8.16 was higher (by >30%) on day 12 than day 15 while under pH 7.65 L production was lower (>50%) on day 12 than day 15 (Table 1).

## Discussion

Our results reveal that local anthropogenic drivers such as copper pollution have a greater impact on kelp meiospore survival and ontogenic development than global climate drivers such as OW and OA. While the independent effects of OW and OA on different early life history stage processes (e.g., meiospore germination and gametophyte growth) are mostly insignificant, the effect of copper is negative and magnified through the different developmental stages. For example, in our experiments, copper exposure (Cu-EC_50_) had only a moderate negative effect (5–18% reduction) on meiospore germination for all OW treatment and the ambient temperature/OA treatment and no effect for the ambient pH/temperature treatment. However, the subsequent growth of germlings was reduced by 43–68%, and sexual differentiation was inhibited regardless of seawater temperature and pH. The different sensitivities of early life history stage processes, not only to copper exposure, but also to other environmental drivers, are related to the fact that meiospore germination is an autogenous process supported by cellular lipid reserves^38–40^ while gametophyte growth and subsequent life-history processes are dependent on the photosynthesis and factors affecting this process^40,41^.

Despite the initial germination process being autogenous, copper exposure can interfere with germ tube initiation in brown seaweeds. In the Fucales, Ca^2+^ movement across the cell membrane of zygotes generates an electrical gradient that initiates the movement of negatively charged vesicles into the basal pole, leading to adhesion and rhizoid formation and germ tube formation^42,43^. An excess of copper may alter Ca^2+^ membrane permeability inhibiting cellular polarization and delay germination in brown macroalgae^42,43^. For example, germination and rhizoid elongation in the brown macroalgae *Fucus serratus* (Order Fucales) was inhibited by copper at 0–2.11 µM Cu^44^ while Cu-EC_50_ for spore germination in *M*. *pyrifera* and *U*. *pinnatifida* are 2.36 and 3.62 µM Cu_T_, respectively. Following the transport of copper into the cytosol, copper disrupts enzyme-active sites and cell division^45,46^. In various organelles, copper interferes with mitochondrial electron transport, respiration, ATP production, and photosynthesis in the chloroplast^47^. The multiple effects of copper in subcellular organelles is likely responsible for the more pronounced effects on germling and gametophyte growth compared to meiospore germination in our experiment.

In general, the production of L seems to be the first line of defence by macroalgae against copper toxicity conferring some degree of tolerance by neutralizing the negative effect of copper^21,24^. In our study, when copper was removed, *M*. *pyrifera* germling growth rate was significantly enhanced under OA regardless of temperature whereas *U*. *pinnatifida* germling growth did not significantly increase under OA, OW or ambient conditions, indicating a more serious disruption of the development of *M*. *pyrifera* germlings under copper stress. In addition, since L cannot to be detected when Cu’ is in excess of CuL^27^, after removing copper from the medium at day 9 onwards, L was detected in cultures of both species. L in the No-Cu treatments was significantly lower than that in Cu-EC_50_ treatment, especially for *U*. *pinnatifida*, suggesting that L release is an active response to Cu stress that enabled the cells to resume growth during the recovery phase. The production of L in response to copper exposure has been reported in adult macroalgae^21,24^, but, to our knowledge, this is the first study showing L production by microscopic early life history stages of the Order Laminariales.

L were not detectable during copper exposure (day 1–9) using our method, but the observed difference between Cu_T_ and Cu’ under No-Cu and Cu-EC_50_ treatment indicates the presence of L, and we suggest that L was already actively produced during that period. The apparent L production may have helped to protect and detoxify kelp cells, thereby promoting germling growth, albeit at very low rates.

Considering that *M*. *pyrifera* and *U*. *pinnatifida* were exposed to different germination Cu-EC_50_ values (2.36 µM for *M*. *pyrifera* and 3.62 µM for *U*. *pinnatifida*^5^), it is noteworthy that growth rate under these Cu-EC_50_ treatments (Fig. 3) was comparable between the two species. However, during recovery (day 12–18), *M*. *pyrifera* germling growth rates were significantly enhanced under OA, regardless of temperature while the growth of *U*. *pinnatifida* germling remained at the same rate at that observed during copper exposure, regardless of pH and temperature treatments. As the Cu-EC_50_ was 35% greater for *U*. *pinnatifida* (3.84 µM Cu_T_) compared to *M*. *pyrifera* (2.51 µM Cu_T_), it is likely that the higher levels of remaining Cu in the *U*. *pinnatifida* cultures adsorbed into the container and/or the cell wall and negatively affected growth rate recovery germlings. In contrast, there was less Cu remaining in the *M*. *pyrifera* cultures and the higher *p*CO_2_ (Table 2) enhanced the growth rate of germlings.

**Table 2:** Carbonate chemistry parameters were calculated from total alkalinity (AT; n = 3) and dissolved inorganic carbon (DIC; n = 3) measurements of seawater (salinity 35‰) corresponding to two temperatures (12 and 16 °C) and two pH (pH_T_ 7.65 and 8.16) treatments.

Temperature	Seawater parameter	Seawater pH_T_ treatments	
		OA	Ambient
12 °C	pH_T_	7.65 ± 0.001	8.16 ± 0.010
	DIC (µmol·Kg^−1^)	2180.12 ± 11.56	2063.04 ± 5.00
	AT (µmol·Kg^−1^)	2230.31 ± 5.16	2236.88 ± 7.01
	HCO_3_^−^ (µmol·Kg^−1^)	2074.25 ± 11.88	1917.65 ± 9.99
	H_2_CO_3_^−^ (µmol·Kg^−1^)	43.78 ± 2.69	18.28 ± 1.10
	CO_3_^2−^ (µmol·Kg^−1^)	62.09 ± 3.12	127.11 ± 6.53
	*p*CO_2_ (µatm)	1074.71 ± 66.06	448.73 ± 27.09
16 °C	pH_T_	7.65 ± 0.004	8.16 ± 0.007
	DIC (µmol·Kg^−1^)	2147.74 ± 4.12	2053.49 ± 5.96
	AT (µmol·Kg^−1^)	2221.72 ± 9.69	2222.25 ± 14.1
	HCO_3_^−^ (µmol·Kg^−1^)	1869.32 ± 9.57	1910.85 ± 13.8
	H_2_CO_3_^−^ (µmol·Kg^−1^)	35.70 ± 3.61	18.35 ± 1.76
	CO_3_^2−^ (µmol·Kg^−1^)	72.81 ± 6.75	124.292 ± 10.80
	*p*CO_2_ (µatm)	871.42 ± 88.1	447.939 ± 42.96

Mean ± SD (n = 3) are reported for each seawater treatment.

The species-specific response to copper toxicity observed for *M*. *pyrifera* and *U*. *pinnatifida* may also be attributed to bacteria that inhabit macroalgal surface. The surface of macroalgae is a nutrient-rich habitat that is optimal for colonization by bacteria^48^. Bacteria associated with macroalgae can also exude L for binding and transport metals required for several physiological processes of bacteria such as nitrogen fixation^49^. Thus, L released by bacteria can play an additional protective role for macroalgae against metal pollution^24,49^. For example, research on *M*. *pyrifera* from California indicated that populations from highly copper-polluted coasts have epibiotic bacteria with greater copper tolerance compared with those from less copper-impacted coasts^50^. Therefore, although bacteria were not observed by light microscopy during our experiment, it is possible that bacteria increased the metal tolerance of different life stages of kelps under copper exposure, but this needs further investigation.

The effects of copper on *M*. *pyrifera* and *U*. *pinnatifida* became apparent at the germling and gametogenesis stages of both species, with the growth rate of germlings being significantly lower and gametophyte development (growth and sexual differentiation) arrested in all temperature and pH treatments when copper was added. Trace metals, including copper, are known to promote oxidative damage by increasing the cellular concentration of reactive oxygen species (ROS) such as the superoxide anion (O_2_^−^), hydrogen peroxide (H_2_O_2_) and the hydroxyl radical (OH^−^), and by disrupting the photosynthetic electron chain and reducing the cellular antioxidant capacity in macroalgae^51,52^. At high concentrations, ROS are toxic to all organisms, oxidizing proteins, lipids and nucleic acids that often lead to structural aberrations, mutagenesis, and cell death^51^. Consequently, the presence of ROS likely resulted in the observed low germination and growth rates under copper exposure. In addition, this response might be related to copper inhibiting the utilization (but not the production) of vesicle-stored reserves, e.g., alginic acid^38,40,53^, as the growth of germlings depends on the formation of the new cell wall that contains alginic acid^54,55^. This suggests that copper was preventing cell expansion and growth of *M*. *pyrifera* and *U*. *pinnatifida* germlings during the Cu-EC_50_ exposure in this study.

The initial Cu_T_ concentrations were reduced during the 9-day Cu-EC_50_ exposure likely due to adsorption into exopolysaccharides. This suggestion is supported by the observations that macro- and microalgae and bacteria, over-produce cell wall polysaccharides in response to trace metal pollution to avoid absorption of metals into the cell^56,57^. The negatively-charged active groups (i.e., hydroxyl, sulphate, and carboxyl) of polysaccharides are strong ion-exchangers, and so have a high capacity to bind (i.e., bioadsorb) trace metal ions such as Cu^2+^ ^56^. The extracellular polysaccharide, alginate (i.e., insoluble salt of alginic acid), occurs naturally in brown macroalgae (Ochrophyta, Phaeophyceae) as a major structural component of the matrix of cell wall^56^. It is possible that developing meiospores (in both No-Cu and Cu-EC_50_ treatments) produced enough alginate to block cellular entry of copper to the cytosol, thereby limiting subcellular toxic effects of copper. This protective mechanism might have been operating during the development of *M*. *pyrifera* and *U*. *pinnatifida* meiospores in our experiment, but to assess this, further studies on the production of cell wall polysaccharides by kelp meiospores during copper exposure are required.

**Figure 6:**
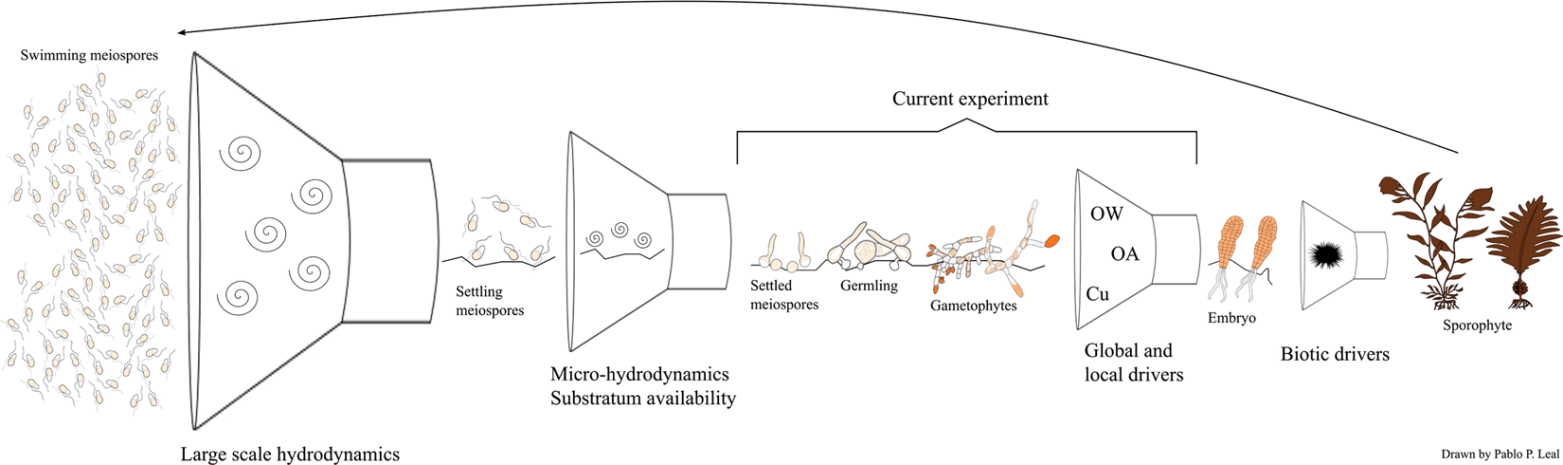
Schematic representation of the bottleneck effect produced by drivers that may influence meiospore settlement and subsequent development into an adult sporophyte population of kelps. Results of the current experiment indicate that early life stages of kelps are susceptible to the interaction between OW, OA and Cu but other drivers may affect the same and different developmental stages. For example, swimming meiospores are affected by large-scale hydrodynamics (e.g., currents) impacting their density and physical transport to the substratum. Micro-hydrodynamics (e.g., small-scale currents) may determine meiospore settlement and subsequent development. Simultaneously, early life history stages of kelps are constantly stressed by the interactions of abiotic (e.g., OW, OA, Cu) and biotic (e.g., grazing) drivers. All these interactions control the dynamic and structure of the adult populations. Adapted from Pineda^66^. Diagram is not drawn to scale.

Millions of meiospores (e.g., >5 × 10^3^ cell mL^−1^ cm of sorus area^−2^) are produced by one fertile sporophyte^29^ so the individual and interactive effects of OW and OA on meiospore germination would be small (15%) and cause little concern. However, upon release, the meiospores are exposed to different abiotic drivers that can already significantly reduce their number before any effect of OW and OA (Fig. 6). These factors include: (1) large-scale hydrodynamics, such as currents affecting the density and the physical transport of the larval pool; (2) micro-hydrodynamics, such as small-scale currents and spatial variability that may determine settlement; and (3) substrate availability and quality, substrate preference and spore settlement behaviour, e.g. phototaxis, chemotaxis^58^. Furthermore, grazing on gametophytes and juvenile sporophytes can further contribute to the decimation and the collapse of the local kelp population^59,60^. The surviving individuals (gametophytes) give rise to the next life history stage (sporophytes) which may be able to withstand exposure to OW and OA due to better acclimation and subsequent adaptation^61^. However, the effect of copper, a relevant local stressor, is more concerning as sexual differentiation and subsequently, sexual reproduction will be arrested further compromising the development of the next generation of sporophytes.

## Methods

### Preparation of trace metal clean, laboratory–ware

All laboratory–ware used for stock solution preparation, seawater sampling and meiospore cultures were acid-cleaned and ultrapure water-rinsed to reduce contamination by trace metals contamination^36^, microalgae and bacteria^24^. Manipulation (e.g., culture media renewal and sampling) during the experiment was performed inside a laminar flow cabinet to minimize contamination^36^.

### Copper stock solution and nominal concentrations

The copper stock solution was prepared by dissolving CuCl_2_ in ultrapure water (2 g L^−1^ CuCl_2_, i.e., 14.9 mM Cu) in a 100-mL polycarbonate bottle (Nalgene^TM^, Nalge Nunc International Corporation, NY, U.S.A.) and stabilized by adding 2 M HCl until reaching pH 2.4^36^. This stock solution was prepared at the beginning of the experiment. The nominal copper concentrations used in this experiment were the theoretical Cu concentration received when diluting the stock solution for each treatment. We aimed for the species-specific Cu_T_ concentrations that inhibits 50% of germinations (Cu-EC_50_ treatment) of 2.36 µM for *M*. *pyrifera* and 3.62 µM for *U*. *pinnatifida*^5^. No-Cu treatment corresponded to the media without any Cu stock addition.

### Total dissolved copper (Cu_T_) analysis

Cu_T_ in the 0.2 µm-filtered solution was measured in the fresh culture media before exposure to the meiospores and at day 0–15. Cu_T_ in the culture media was determined from two replicates of each copper treatment and the analytical blanks. An amount of 0.15 mL of culture medium was diluted in 4.25 mL of ultrapure water and acidified with 0.10 mL of HNO_3_ (acidified sample, 4.5 mL 2% HNO_3_) and stored until analysis. Total copper concentrations were quantified by inductively coupled plasma mass spectrometry (ICP-MS)^5^.

### Labile copper (Cu’) and copper-binding ligand (L) analyses

Cu’ and L in solution were measured from the meiospore culture media on day 0–15. Due to a large number of samples and time requirements for each analysis, Cu’ and natural L concentrations were determined from one sample of each copper treatment by cathodic stripping voltammetry (CSV) of freshly thawed samples at the ambient pH. Good reproducibility was demonstrated for one sample (i.e., *U*. *pinnatifida*, 12 °C and pH_T_ 7.65) on day 6. Due to the limited seawater volumes available, the large number of samples and the fact that most of the samples contained high amounts of copper (i.e., in excess of natural L), a kinetic approach as described for Fe with 1-nitroso-2-naphthol in Witter and Luther III^27^ was used here for Cu with salicylaldoxime (SA) as the competing L^62^. Briefly, to determine Cu’, [CuL] and therefore [L], SA was added to a final concentration of 100 µmol L^−1^. The current was monitored immediately after the addition of SA and until the system reached equilibrium, which was reached at a maximum of 2 h. The current measured right after the addition of SA represents the Cu’ and the difference after 2 h additionally include [CuL]. This assumes that due to the large excess of SA over L, any Cu’ formed during the dissociation of CuL will react faster with SA than with L, and the product CuSAx will not revert to Cu’ during the timescale of the analysis^27^. At the end of the equilibration time a two-fold standard addition was performed to derive the Cu’ concentration. The standard addition curves were in all cases linear, indicating that no free L was present at that time. For samples at Cu-EC_50_ concentrations, no CuL could be detected, i.e. there was no significant difference between the current measured right after the addition of SA and after equilibration of 2 h. However, we could still derive Cu’ from our analysis. For No-Cu treatments and samples taken during the recovery period, i.e. 9–15 days, the current increased during the equilibration time and [CuL] could be approximated. The errors associated with the analysis, however, were too large to derive meaningful stability constants from these measurements and we therefore report only the L concentrations.

### Sampling location and sporophyll collection

*M*. *pyrifera* and *U*. *pinnatifida* can be found cohabiting in the shallow subtidal zone in Hamilton Bay (45°47′51″S; 170°38′39″E), as well as in other bays within the Otago Harbour, New Zealand^63^. The surface seawater temperature in the Otago Harbour varies between 6 and 18 °C annually, with seasonal ranges of 15 to 18 °C in summer, 10 to 16 °C in autumn, 6 to 9 °C in winter, and 10 to 15 °C in spring^64,65^. Sporophylls with fertile tissue (i.e., sori) were collected from ten adult sporophytes of *M*. *pyrifera* and *U*. *pinnatifida* during low tide from the upper sub-littoral zone of Hamilton Bay in November 2014 (Southern Hemisphere’ spring). In the laboratory, collected sporophylls were lightly brushed and cleaned of visible epibiota under filtered (0.2 µm, Whatman™ Polycap™ TC filter capsule, GE Healthcare Life Sciences, UK) seawater, blotted dry, wrapped in tissue paper and kept overnight at 4 °C to induce dehydration before meiospore release.

### Seawater pH measurements

The seawater pH during the experiment was measured on the total scale (pH_T_) at 12 and 16 °C using a pH electrode (Orion ROSS Sure-Flow semi-micro, ORI8175BNWPW) connected to a pH meter (Thermo Scientific Orion 720 A pH/ION Meter). The electrode slope was determined using temperature equilibrated pH 7 and pH 9 buffers (colour coded, NIST traceable). pH was measured on the total scale using TRIS and 2-aminopyridine buffers in synthetic seawater to calibrate the electrode^66^. Seawater samples representing the two pH_T_ treatments were collected and fixed with mercuric chloride for determining seawater carbonate chemistry. Total alkalinity (AT) was measured using the closed-cell titration method and dissolved inorganic carbon (DIC) was measured directly by acidifying the sample^66^. The seawater carbonate chemistry of each pH treatment at both temperatures was calculated using the measured AT, DIC, pH, salinity, and temperature (Table 2) with the SWCO2 software^67^.

### Seawater pH treatments

The seawater used in the experiment was collected at the same time as the sporophylls and had a salinity of 36‰. The seawater was filtered (0.2 µm) to reduce microalgal and bacterial contamination and kept overnight in previously sterilized 2 L-polycarbonate bottles at the respective temperature treatment before use. After filtration, nutrients (10 µM NaNO_3_ and 1 µM NaH_2_PO_4_) were added to the seawater to avoid nutrient limitation. The present pH treatment corresponded to the non-manipulated seawater (pH_T_ 8.16, defined as ambient treatment). To obtain the lowest seawater pH treatment (pH_T_ 7.65, defined as OA treatment), equal volumes of 0.5 M HCl and 0.5 M NaHCO_3_ were added to the seawater^68,69^ until pH_T_ reached 7.65 at 16 °C. Seawater with the corresponding pH_T_ was freshly prepared every three days to renew the culture medium.

### Effects of seawater temperature, pH, and copper on meiospore development

Meiospore release and cultivation were performed as described in Leal *et al*.^32^. Briefly, from each species, discs (2 cm^2^) of mature sorus were cut from the sporophylls using a cork borer. Pools of excised sori (total of ca. 50 g of 2 cm^2^ discs each) of both kelp species were separately immersed in seawater of the different pH_T_ and temperature treatments for 15 min. After release, meiospores were dispensed (final density of 25,000 cell·mL^−1^) into culture flasks (Corning® 75 cm^2^, polystyrene cell culture flask with phenolic-style cap) containing seawater with the corresponding pH_T_ and temperature but without copper addition. To avoid meiospore mortality during swimming and settlement that could change the initial density, meiospores were allowed to settle for 3 h before exposure to the respective nutrient-amended seawater and copper concentration treatments. Exposure to copper lasted 9 days, which is the time observed to inhibit gametogenesis in both kelp species^5^. Meiospore cultures of both species under the respective pH_T_ and copper treatments were prepared in two sets and each one was exposed to the respective temperature treatment (12 and 16 °C) in two identical temperature-controlled chambers (Contherm Plant Growth Chamber, Contherm Scientific Co. Ltd., New Zealand). PAR (metal halide lamps Philips HPI- T 400 W quartz), with a photoperiod of 12 h light: 12 h dark, was measured with a spherical quantum sensor (LI-193, LI-COR, Lincoln, Nebraska) connected to a light meter (LI-250A, LI-COR, Lincoln, Nebraska) and adjusted to 55 ± 2 and 54 ± 1 µmol photons·m^−2^ s^−1^ in the 12 °C- and 16 °C-culture room, respectively. Thereafter, Cu-treated samples were allowed to recover using the culture medium with nutrients but without copper addition, under the respective scenario. Analytical blanks (i.e., seawater with each copper concentration under the respective pH and temperature conditions but without biological material) corresponding to each copper treatment were also prepared. The media of the meiospore cultures with the appropriate pH_T_, copper, and nutrients (to avoid nutrient depletion), were renewed every 3 days. Cu_T_ concentrations in the treatments and blanks were measured as described above.

### Meiospore development

*M*. *pyrifera* and *U*. *pinnatifida* meiospore germination (%), germling growth rate (%·day^−1^), gametophyte size (µm^2^) and sex ratio, during the experiment, were obtained from photograph (5.1 M CMOS camera, UCMOS0510KPA) taken every three days from at least five haphazardly chosen visual fields, using an inverted microscope (200×, Olympus CK2; Olympus Optical Co. Ltd., Tokyo, Japan). Photographs were analysed using the ToupView 3.5 digital camera software (ToupTek Photonics, Zhejiang, China). Meiospores with visible germ tubes were considered germinated and the germination percentage was calculated from 350 individuals per replicate after 6 d of culture. The size of sexually ambiguous growing meiospores (germlings) and sexually-differentiated male and female gametophytes was obtained from an average of 30 individuals per replicate after 12 and 15 d of culture, respectively. Germling growth rate under No-Cu and Cu-EC_50_ treatments were separately calculated during exposure and recovery. For germlings under No-Cu treatment, growth rate was calculated from 0 to 12 d, before sexual differentiation was observed in both kelps. For germlings under Cu-EC_50_ treatment, growth rate was calculated during copper exposure (0–9 d) and during recovery period (12–18 d). Growth rate (%·day^−1^) was calculated as [(*W*_*t*_/*W*_0_)^1/*t*^−1] × 100, where *W*_0_ is the initial size, *W*_*t*_ is the final size, and *t* is days of culture^70^. At day 15, when sexual ambiguity was resolved, male and female gametophytes were counted and the sex ratio, expressed as the frequency of males per progeny, was calculated as no. ♂/(no. ♂ + no. ♀)^69^.

### Statistical analyses

We did not statistically compare the two species in the present work because *M*. *pyrifera* and *U*. *pinnatifida* showed species-specific responses to copper^5^, OA and/or OW^32,33^ in previous studies. Percentage germination and germling growth rate (%·day^−1^) were logit transformed^71^. All the data satisfied Normality (Kolgomorov-Smirnov test) and homogeneity of variances (Levene’s test). Three-way ANOVA (*P* < 0.05) was used to test the statistical significance of differences in meiospore germination, germling growth rate during copper exposure, gametophyte size, germling growth rate (copper exposure vs. recovery period) between temperature, pH, copper treatments, and sex. Two-way ANOVA (*P* < 0.05) was used to test the statistical significance of differences in gametophyte sex ratio and germling growth rate (during recovery) between temperature and pH. When significant interactive effects were observed in the ANOVAs (at α = 0.05), the significant main effects of the factors (i.e., temperature, pH, copper treatments and sex) were subordinated, and the interaction(s) becomes the focus of the analysis^72^. A post hoc Tukey test (*P* < 0.05) was applied when a significant effect (single, two- and/or three-way interactions) of independent variables was observed. The ANOVA analyses were run using the software SigmaPlot version 12.0 (Systat Software, Inc., San Jose, CA). ANOVA statistical results for *M*. *pyrifera* and *U*. *pinnatifida* are listed in Supplementary Tables S1 and S2, respectively.

## Electronic supplementary material

Supplementary Information

## References

[1] IPCC. *Climate change 2013: the physical science basis*. *Contribution of working group I to the fifth assessment report of the intergovernmental panel on climate change*. (Cambridge University Press, Cambridge, United Kingdom and New York, 2013).

[2] Koch M, Bowes G, Ross C, Zhang XH (2013). Climate change and ocean acidification effects on seagrasses and marine macroalgae. Glob. Chang. Biol..

[3] Halpern BS (2008). A global map of human impact on marine ecosystems. Science (80-).

[4] Gledhill M, Nimmo M, Stephen JH, Brown MT (1997). The toxicity of copper(II) species to marine algae, with particular reference to macroalgae. J. Phycol..

[5] Leal PP, Hurd CL, Sander SG, Kortner B, Roleda MY (2016). Exposure to chronic and high dissolved copper concentrations impede meiospore development of the kelps *Macrocystis pyrifera* and *Undaria pinnatifida* (Ochrophyta). Phycologia.

[6] Millero FJ, Woosley R, DiTrolio BR, Waters J (2009). Effects of the ocean acidification on the speciation of metals in seawater. Oceanography.

[7] Zeng X, Chen X, Zhuang J (2015). The positive relationship between ocean acidification and pollution. Mar. Pollut. Bull..

[8] Abualhaija MM, Whitby H, van den Berg CMG (2015). Competition between copper and iron for humic ligands in estuarine waters. Mar. Chem..

[9] Worms I, Simon DF, Hassler CS, Wilkinson KJ (2006). Bioavailability of trace metals to aquatic microorganisms: importance of chemical, biological and physical processes on biouptake. Biochimie.

[10] Richard Y (2013). Temperature changes in the mid-and high-latitudes of the Southern Hemisphere. Int. J. Climatol..

[11] Russell BD, Connell SD (2012). Origins and consequences of global and local stressors: incorporating climatic and non-climatic phenomena that buffer or accelerate ecological change. Mar. Biol..

[12] Clayson CA, Bogdanoff AS (2013). The effect of diurnal sea surface temperature warming on climatological air–sea fluxes. J. Clim..

[13] Large WG, Caron JM (2015). Diurnal cycling of sea surface temperature, salinity, and current in the CESM coupled climate model. J. Geophys. Res. Ocean..

[14] Hofmann GE (2011). High-frequency dynamics of ocean pH: a multi-ecosystem comparison. Plos One.

[15] Cornwall CE (2013). Diurnal fluctuations in seawater pH influence the response of a calcifying macroalga to ocean acidification. Proc. R. Soc. B.

[16] Lewis, A. G. *Copper in water and aquatic environment*s (1995).

[17] Nor YM (1987). Ecotoxicity of copper to aquatic biota: a review. Environ. Res..

[18] Correa JA (1999). Copper, copper mine tailings and their effect on marine algae in Northern Chile. J. Appl. Phycol..

[19] Raven JA (2003). Inorganic carbon concentrating mechanisms in relation to the biology of algae. Photosynth. Res.

[20] Krämer, U. & Clemens, S. Functions and homeostasis of zinc, copper, and nickel in plants. In *Molecular Bology of Metal Homeostasis and Detoxification from Microbes to* Man (eds Tamás, M. J. & Martinoia, E.) **14**, 214–272 (Springer-Verlag, 2006).

[21] Murray H, Meunier G, van den Berg CMG, Cave RR, Stengel DB (2014). Voltammetric characterisation of macroalgae-exuded organic ligands (L) in response to Cu and Zn: a source and stimuli for L. Environ. Chem..

[22] Croot PL, Moffett JW, Brand LE (2000). Production of extracellular Cu complexing ligands by eukaryotic phytoplankton in response to Cu stress. Limnol. Oceanogr..

[23] Sueur S, van den Berg CMG, Riley JP (1982). Measurement of the metal complexing ability of exudates of marine macroalgae. Limnol. Oceanogr..

[24] Gledhill M, Nimmo M, Hill SJ, Brown MT (1999). The release of copper-complexing ligands by the brown alga *Fucus vesiculosus* (Phaeophyceae) in response to increasing total copper levels. J. Phycol..

[25] Vasconcelos MTSD, Leal MFC (2001). Seasonal variability in the kinetics of Cu, Pb, Cd and Hg accumulation by macroalgae. Mar. Chem..

[26] Bruland KW, Rue EL, Donat JR, Skrabal SA, Moffett JW (2000). Intercomparison of voltammetric techniques to determine the chemical speciation of dissolved copper in a coastal seawater sample. Anal. Chim. Acta.

[27] Witter AE, Luther GW (1998). Variation in Fe-organic complexation with depth in the Northwestern Atlantic Ocean as determined using a kinetic approach. Mar. Chem..

[28] Witter AE, Hutchins DA, Butler A, Luther GW (2000). Determination of conditional stability constants and kinetic constants for strong model Fe-binding ligands in seawater. Mar. Chem..

[29] Leal PP, Hurd CL, Roleda MY (2014). Meiospores produced in sori of non-sporophyllous laminae of *Macrocystis pyrifera* (Laminariales, Phaephyceae) may enhance reproductive output. J. Phycol..

[30] Xie ZC, Nga CW, Qian PY, Qiu JW (2005). Responses of polychaete *Hydroides elegans* life stages to copper stress. Mar. Ecol. Prog. Ser..

[31] Nielsen SL, Nielsen HD, Pedersen MF (2014). Juvenile life stages of the brown alga *Fucus serratus* L. are more sensitive to combined stress from high copper concentration and temperature than adults. Mar. Biol..

[32] Leal PP, Hurd CL, Fernández PA, Roleda MY (2017). Ocean acidification and kelp development: reduced pH has no negative effects on meiospore germination and gametophyte development of *Macrocystis pyrifera* and *Undaria pinnatifida*. J. Phycol..

[33] Leal PP, Hurd CL, Fernández PA, Roleda MY (2017). Meiospore development of the kelps *Macrocystis pyrifera* and *Undaria pinnatifida* under ocean acidification and ocean warming: independent effects are more important than their interaction. Mar. Biol..

[34] Campbell AL, Mangan S, Ellis RP, Lewis C (2014). Ocean acidification increases copper toxicity to the early life-history stages of the polychaete *Arenicola marina* in artificial seawater. Environ. Sci. Technol..

[35] Roberts DA (2013). Ocean acidification increases the toxicity of contaminated sediments. Glob. Chang. Biol..

[36] Leal PP, Hurd CL, Sander SG, Armstrong EA, Roleda MY (2016). Copper ecotoxicology of marine algae: a methodological appraisal. Chem. Ecol..

[37] Roleda MY (2015). Effect of ocean acidification and pH fluctuations on the growth and development of coralline algal recruits, and an associated benthic algal assemblage. Plos One.

[38] Brzezinski MA, Reed DC, Amsler CD (1993). Neutral lipids as major storage products in zoospores of the giant kelp Macrocystis pyrifera (Phaeophyceae). J. Phycol..

[39] Reed DC, Brzezinski MA, Coury DA, Graham WM, Petty RL (1999). Neutral lipids in macroalgal spores and their role in swimming. Mar. Biol..

[40] Steinhoff FS, Graeve M, Wiencke C, Wulff A, Bischof K (2011). Lipid content and fatty acid consumption in zoospores/developing gametophytes of *Saccharina latissima* (Laminariales, Phaeophyceae) as potential precursors for secondary metabolites as phlorotannins. Polar Biol..

[41] Amsler CD, Neushul M (1991). Photosynthetic physiology and chemical composition of spores of the kelps *Macrocystis pyrifera*, *Nereocystis luetkeana*, *Laminaria farlowii*, and *Pterygophora californica* (Phaeophyceae). J. Phycol..

[42] Anderson BS, Hunt JW, Turpen SL, Coulon AR, Martin M (1990). Copper toxicity to microscopic stages of giant kelp *Macrocystis pyrifera*: interpopulation comparisons and temporal variability. Mar. Ecol. Prog. Ser..

[43] Burridge TR, Portelli T, Ashton P (1996). Effect of sewage effluents on germination of three marine brown algal macrophytes. Mar. Freshw. Res..

[44] Nielsen HD, Brown MT, Brownlee C (2003). Cellular responses of developing *Fucus serratus* embryos exposed to elevated concentrations of Cu. Plant, Cell Environ..

[45] Stauber JL, Florence TM (1985). Interactions of copper and manganese: a mechanism by which manganese alleviates copper toxicity to the marine diatom, *Nitzschia closterium* (Ehrenberg) W. Smith. Aquat. Toxicol..

[46] Florence TM, Stauber JL (1986). Toxicity of copper complexes to the marine diatom *Nitzschia closterium*. Aquat. Toxicol..

[47] Stauber JL, Florence TM (1987). Mechanism of toxicity of ionic copper and copper complexes to algae. Mar. Biol..

[48] Armstrong EA, Yan L, Boyd KG, Wright PC, Burgess JG (2001). The symbiotic role of marine microbes on living surfaces. Hydrobiologia.

[49] Wichard T (2016). Identification of metallophores and organic ligands in the chemosphere of the marine macroalga *Ulva* (Chlorophyta) and at land-sea interfaces. Front. Mar. Sci..

[50] Busch J, Nascimento JR, Magalhães ACR, Dutilh BE, Dinsdale E (2015). Copper tolerance and distribution of epibiotic bacteria associated with giant kelp *Macrocystis pyrifera* in southern California. Ecotoxicology.

[51] Pinto E (2003). Heavy metal-induced oxidative stress in algae. J. Phycol..

[52] Collén J, Pinto E, Pedersén M, Colepicolo P (2003). Induction of oxidative stress in the red macroalga *Gracilaria tenuistipitata* by pollutant metals. Arch. Environ. Contam. Toxicol..

[53] Reed DC, Amsler CD, Ebeling AW (1992). Dispersal in kelps: factors affecting spore swimming and competency. Ecology.

[54] Bond PR (1999). Arrested development in Fucus spiralis (Phaeophyceae) germlings exposed to copper. Eur. J. Phycol..

[55] Brawley SH, Wetherbee R, Quatrano RS (1976). Fine-structural studies of the gametes and embryo of *Fucus vesiculosus* L.(Phaeophyta). II. The cytoplasm of the egg and young zygote. J. Cell Sci..

[56] Davis TA, Volesky B, Mucci A (2003). A review of the biochemistry of heavy metal biosorption by brown algae. Water Res..

[57] Hay ID, Rehman ZU, Moradali MF, Wang Y, Rehm BHA (2013). Microbial alginate production, modification and its applications. Microb. Biotechnol..

[58] Pineda J (2000). Linking larval settlement to larval transport: assumptions, potentials, and pitfalls. Oceanogr. East. Pacific.

[59] Reed D (2016). Extreme warming challenges sentinel status of kelp forests as indicators of climate change. Nat. Commun..

[60] Schiel DR, Foster MS (2006). The population biology of large brown seaweeds: ecological consequences of multiphase life histories in dynamic coastal environments. Annu. Rev. Ecol. Evol. Syst..

[61] Harley CDG (2012). Effects of climate change on global seaweed communities. J. Phycol..

[62] Campos MLAM, van den Berg CMG (1994). Determination of copper complexation in sea water by cathodic stripping voltammetry and ligand competition with salicylaldoxime. Anal. Chim. Acta.

[63] Russell LK, Hepburn CD, Hurd CL, Stuart MD (2008). The expanding range of *Undaria pinnatifida* in southern New Zealand: distribution, dispersal mechanisms and the invasion of wave-exposed environments. Biol. Invasions.

[64] Brown MT, Nyman MA, Keogh JA, Chin NKM (1997). Seasonal growth of the giant kelp *Macrocystis pyrifera* in New Zealand. Mar. Biol..

[65] Kregting LT, Hepburn CD, Hurd CL, Pilditch CA (2008). Seasonal patterns of growth and nutrient status of the macroalga *Adamsiella chauvinii* (Rhodophyta) in soft sediment environments. J. Exp. Mar. Bio. Ecol.

[66] Dickson, A. G., Sabine, C. L. & Christian, J. R. *Guide to Best Practices for Ocean CO2 Measurement*s (2007).

[67] Hunter, K. A. SWCO2. Available at, http://neon.otago.ac.nz/research/mfc/people/keith_hunter/software/swco2. (Accessed: 1st May 2015) (2007).

[68] Riebesell, U., Fabry, V. J., Hansson, L. & Gattuso, J.-P. *Guide to best practices for ocean acidification research*. (Publications Office of the European Union, 2010).

[69] Roleda MY, Morris JN, McGraw CM, Hurd CL (2012). Ocean acidification and seaweed reproduction: increased CO ameliorates the negative effect of lowered pH on meiospore germination in the giant kelp *Macrocystis pyrifera* (Laminariales, Phaeophyceae). Glob. Chang. Biol..

[70] Yong YS, Yong WTL, Anton A (2013). Analysis of formulae for determination of seaweed growth rate. J. Appl. Phycol..

[71] Warton DI, Hui FK (2011). The arcsine is asinine: the analysis of proportions in ecology. Ecology.

[72] Sokal, R. R. & Rohlf, F. J. *Biometry: the principles and practice of statistics in biological research*. (W. H. Freeman and Co., 2012).

