# Supplementary material for: Copper pollution exacerbates the effects of ocean acidification and warming on kelp microscopic early life stages
Source: Sci Rep. 2018 Oct 3;8:14763. doi: 10.1038/s41598-018-32899-w (PMC6170414; doi:10.1038/s41598-018-32899-w)
Supplement: Supplementary file 1 — Supplementary Information [file 41598_2018_32899_MOESM1_ESM.docx]

**Copper pollution exacerbates the effects of ocean acidification and warming on kelp microscopic early life stages**

Pablo P. Leal^1,2,3,^*, Catriona L Hurd^2^, Sylvia G. Sander^4,5^, Evelyn Armstrong^4^, Pamela A. Fernández^6^, Tim J. Suhrhoff^7^, Michael Y. Roleda^1,8,9^

^1^Department of Botany, University of Otago, 479 Great King Street, Dunedin 9016, New Zealand.

^2^Institute for Marine and Antarctic Studies, University of Tasmania, 20 Castray Esplanade Battery Point, Hobart 7004, Tasmania.

^3^Departamento de Repoblación y Cultivo, Instituto de Fomento Pesquero (IFOP), Balmaceda 252, Puerto Montt, Casilla 665, Chile.

^4^National Institute for Water and Atmospheric (NIWA)/University of Otago Research Centre for Oceanography, Union Place West, Dunedin 9016, New Zealand.

^5^Marine Environment Study Laboratory, International Atomic Energy Agency, 4 Quai Antione 1er, 98000 Monaco, Principality of Monaco.

^6^Centro i~mar, Universidad de Los Lagos, Camino a Chinquihue Km 6, Puerto Montt, Casilla 557, Chile.

^7^ETH Zürich, Institute of Geochemistry and Petrology, Department of Earth Sciences, Clausiusstrasse 25, 8092, Zürich, Switzerland.

^8^Norwegian Institute of Bioeconomy Research, Kudalsveien 6, 8027 Bodø, Norway.

^9^The Marine Science Institute, College of Science, University of the Philippines Diliman, Quezon City, Philippines.

*[pablo.leal@ifop.cl](mailto:pablo.leal@ifop.cl)

**Supplementary Figure S1.** Growth of germlings of *M. pyrifera* and (b) *U. pinnatifida* after 18 days of cultivation in a factorial combination of two temperatures (12 and 16 °C), two pH (pH_T_ 7.65 and 8.16) and two copper (No-Cu, and Cu-EC_50_ = 2.36 and 3.62 µM Cu for *M. pyrifera* and *U. pinnatifida*, respectively) treatments. No data is available for germling size for the No-Cu treatment after day 12 as gametophyte sexual differentiation occurred at day 15. Bars represent mean ± SD (n = 4).


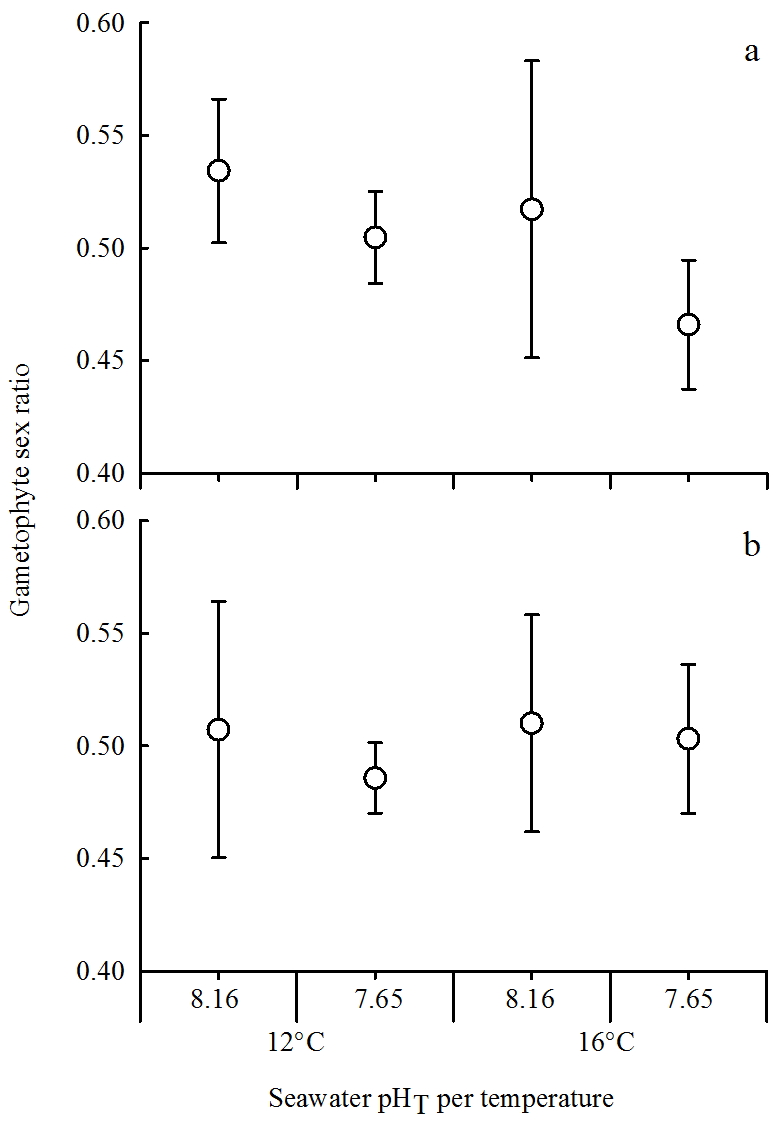


**Supplementary Figure S2.** Sex ratio between male and female gametophytes for (a) *M. pyrifera* and (b) *U. pinnatifida* at the 15^th^ day of culture in a factorial combination of two temperatures (12 and 16 °C) and two pH (pH_T_ 7.65 and 8.16) treatments but only under the No-Cu condition because gametogenesis was not observed under the Cu-EC_50_ condition. Bars represent mean ± SD (n = 4). Note that the y-axis starts at 0.40 in both graphs.

| Variable | Source of variation | Degree of freedom | Sum of squares | *F* | *P* |
| --- | --- | --- | --- | --- | --- |
| Meiospore germination   (Fig. 2a) | Temperature | 1 | 0.117 | 1.647 | 0.212 |
|  | pH_T_ | 1 | 0.119 | 1.677 | 0.208 |
|  | Copper | 1 | 1.885 | 26.543 | < 0.001 |
|  | Temperature × pH_T_ | 1 | 0.478 | 6.726 | 0.016 |
|  | Temperature × copper | 1 | 0.267 | 3.755 | 0.064 |
|  | pH_T_ × copper | 1 | 0.211 | 2.976 | 0.097 |
|  | Temperature × pH_T_ × copper | 1 | 0.192 | 2.703 | 0.113 |
|  | Residual | 24 | 1.704 |  |  |
|  | Total | 31 | 4.973 |  |  |
| Germling growth rate   (Fig. 3a) | Temperature | 1 | 0.0268 | 18.968 | < 0.001 |
|  | pH_T_ | 1 | 0.0157 | 11.100 | 0.003 |
|  | Copper | 1 | 1.298 | 916.693 | < 0.001 |
|  | Temperature × pH_T_ | 1 | 0.00401 | 2.833 | 0.105 |
|  | Temperature × copper | 1 | 0.0000149 | 0.0105 | 0.919 |
|  | pH_T_ treatment × copper | 1 | 0.0274 | 19.352 | < 0.001 |
|  | Temperature × pH_T_ × copper | 1 | 0.00562 | 3.972 | 0.058 |
|  | Residual | 24 | 0.0340 |  |  |
|  | Total | 31 | 1.411 |  |  |
| Gametophyte size   (Fig. 4a) | Temperature | 1 | 75.541 | 0.00125 | 0.972 |
|  | pH_T_ | 1 | 333525.988 | 5.511 | 0.027 |
|  | Sex | 1 | 3878815.346 | 64.087 | < 0.001 |
|  | Temperature × pH_T_ | 1 | 743885.448 | 12.291 | 0.002 |
|  | Temperature × sex | 1 | 42020.052 | 0.694 | 0.413 |
|  | pH_T_ × sex | 1 | 931.606 | 0.0154 | 0.902 |
|  | Temperature × pH_T_ × sex | 1 | 119308.100 | 1.971 | 0.173 |
|  | Residual | 24 | 1452583.082 |  |  |
|  | Total | 31 | 6571145.164 |  |  |
| Gametophyte sex ratio   (Fig. S1a) | Temperature | 1 | 0.0031 | 1.882 | 0.195 |
|  | pH_T_ | 1 | 0.00652 | 3.951 | 0.070 |
|  | Temperature × pH_T_ | 1 | 0.000472 | 0.286 | 0.603 |
|  | Residual | 12 | 0.0198 |  |  |
|  | Total | 15 | 0.0299 |  |  |
| Germling growth rate during recovery period (Fig. 5a) | Temperature | 1 | 0.00599 | 1.079 | 0.319 |
|  | pH_T_ | 1 | 0.0359 | 6.475 | 0.026 |
|  | Temperature × pH_T_ | 1 | 0.0155 | 2.786 | 0.121 |
|  | Residual | 12 | 0.0666 |  |  |
|  | Total | 15 | 0.124 |  |  |
| Germling growth rate  (Comparison between growth rate during day 1 – 9 [copper exposure, Fig. 3a] and during 12 – 18 [recovery period, Fig. 5a]) | Period | 1 | 0.0869 | 22.904 | < 0.001 |
|  | Temperature | 1 | 0.0181 | 4.782 | 0.039 |
|  | pH_T_ | 1 | 0.0130 | 3.422 | 0.077 |
|  | Period × Temperature | 1 | 0.000639 | 0.168 | 0.685 |
|  | Day × pH_T_ | 1 | 0.0238 | 6.259 | 0.020 |
|  | Temperature × pH_T_ | 1 | 0.00879 | 2.316 | 0.141 |
|  | Period × Temperature × pH_T_ | 1 | 0.00674 | 1.776 | 0.195 |
|  | Residual | 24 | 0.0911 |  |  |
|  | Total | 31 | 0.249 |  |  |

**Supplementary Table S1.** ANOVA and significance values for effects of temperature, pH_T_ and copper treatments and their interactions on meiospore germination, germling growth rate, gametophyte size and gametophyte sex ratio of *M. pyrifera*.

| Variable | Source of variation | Degree of freedom | Sum of squares | *F* | *P* |
| --- | --- | --- | --- | --- | --- |
| Meiospore germination   (Fig. 2b) | Temperature | 1 | 0.130 | 4.109 | 0.054 |
|  | pH_T_ | 1 | 0.049 | 1.547 | 0.226 |
|  | Copper | 1 | 0.925 | 29.236 | < 0.001 |
|  | Temperature × pH_T_ | 1 | 0.0275 | 0.870 | 0.360 |
|  | Temperature × copper | 1 | 0.433 | 13.675 | 0.001 |
|  | pH_T_ × copper | 1 | 0.0226 | 0.715 | 0.406 |
|  | Temperature × pH_T_ × copper | 1 | 0.120 | 3.795 | 0.063 |
|  | Residual | 24 | 0.759 |  |  |
|  | Total | 31 | 2.467 |  |  |
| Germling growth rate   (Fig. 3b) | Temperature | 1 | 0.0107 | 3.670 | 0.067 |
|  | pH_T_ treatment | 1 | 0.00313 | 1.075 | 0.310 |
|  | Copper | 1 | 1.686 | 579.507 | < 0.001 |
|  | Temperature × pH_T_ | 1 | 0.00651 | 2..237 | 0.148 |
|  | Temperature × copper | 1 | 0.0207 | 7.131 | 0.013 |
|  | pH_T_ × copper | 1 | 0.00116 | 0.400 | 0.533 |
|  | Temperature × pH_T_ × copper | 1 | 0.000170 | 0.0583 | 0.811 |
|  | Residual | 24 | 0.0698 |  |  |
|  | Total | 31 | 1.798 |  |  |
| Gametophyte size   (Fig. 4b) | Temperature | 1 | 105065.095 | 2.768 | 0.109 |
|  | pH_T_ | 1 | 173836.248 | 4.580 | 0.043 |
|  | Sex | 1 | 25047.547 | 0.660 | 0.425 |
|  | Temperature × pH_T_ | 1 | 23186.678 | 0.611 | 0.442 |
|  | Temperature × sex | 1 | 134081.476 | 3.533 | 0.072 |
|  | pH_T_ × sex | 1 | 35161.833 | 0.926 | 0.345 |
|  | Temperature × pH_T_ × sex | 1 | 15941.956 | 0.420 | 0.523 |
|  | Residual | 24 | 910886.930 |  |  |
|  | Total | 31 | 1423207.763 |  |  |
| Gametophyte sex ratio   (Fig. S1b) | Temperature | 1 | 0.000411 | 0.238 | 0.635 |
|  | pH_T_ | 1 | 0.000804 | 0.466 | 0.508 |
|  | Temperature × pH_T_ | 1 | 0.000209 | 0.121 | 0.734 |
|  | Residual | 12 | 0.0207 |  |  |
|  | Total | 15 | 0.0221 |  |  |
| Germling growth rate during recovery period (Fig. 5b) | Temperature | 1 | 0.0391 | 1.932 | 0.190 |
|  | pH_T_ | 1 | 0.0493 | 2.434 | 0.145 |
|  | Temperature × pH_T_ | 1 | 0.0167 | 0.825 | 0.382 |
|  | Residual | 12 | 0.243 |  |  |
|  | Total | 15 | 0.348 |  |  |
| Germling growth rate  (Comparison between growth rate during day 1 – 9 [copper exposure, Fig. 3b] and during 12 – 18 [recovery period, Fig. 5b]) | Period | 1 | 0.0158 | 1.342 | 0.258 |
|  | Temperature | 1 | 0.0143 | 1.217 | 0.281 |
|  | pH_T_ | 1 | 0.0214 | 1.819 | 0.190 |
|  | Period × Temperature | 1 | 0.0257 | 2.187 | 0.152 |
|  | Period × pH_T_ | 1 | 0.0282 | 2.402 | 0.134 |
|  | Temperature × pH_T_ | 1 | 0.0191 | 1.628 | 0.214 |
|  | Period × Temperature × pH_T_ | 1 | 0.00199 | 0.169 | 0.684 |
|  | Residual | 24 | 0.282 |  |  |
|  | Total | 31 | 0.408 |  |  |

**Supplementary Table S2.** ANOVA and significance values for effects of temperature, pH_T_ and copper treatments and their interactions on meiospore germination, germling growth rate, gametophyte size and gametophyte sex ratio of *U. pinnatifida*.
